# Supplementary material for: Profiling of Epigenetic Features in Clinical Samples Reveals Novel Widespread Changes in Cancer
Source: Cancers (Basel). 2019 May 24;11(5):723. doi: 10.3390/cancers11050723 (PMC6562406; doi:10.3390/cancers11050723)
Supplement: Supplementary file 1 [file cancers-11-00723-s001.zip › cancers-484721-supplementary/cancers-484721-suppl-final.docx]

Supplementary materials

**Table S1.** Summary of breast samples.

| **Cancer Type** | **Subtype** | **Patient Number** | **Sample Type** | | **Immunoprofile^1^** | | | **Ki67 (%)** | **Storage** |
| --- | --- | --- | --- | --- | --- | --- | --- | --- | --- |
|  |  |  | **Normal** | **Tumor** | **ER (%)** | **PR (%)** | **HER2 score** |  |  |
| Breast cancer | Luminal A-like | 1 | normal 1 | tumor 1 | 90 | 90 | Neg. | 10 | fresh-frozen |
|  |  | 2 | normal 2 | tumor 2 | 95 | 95 | Neg. | 15 | fresh-frozen |
|  |  | 3 | normal 3 | tumor 3 | 95 | 95 | Neg. | 18 | fresh-frozen |
|  |  | 4 | normal 4 | tumor 4 | 90 | 90 | Neg. | 19 | fresh-frozen |
|  |  | 5 | normal 5 | tumor 5 | 95 | 95 | Neg. | 13 | fresh-frozen |
|  |  | 6 | normal 6 | tumor 6 | 90 | 90 | Neg. | 15 | OCT-frozen |
|  |  | 7 | normal 7 | tumor 7 | 95 | 95 | Neg. | 16 | OCT-frozen |
|  |  | 8 | normal 8 |  |  |  |  |  | OCT-frozen |
|  |  | 9 |  | tumor 9 | 80 | 80 | Neg. | 19 | OCT-frozen |
|  | Triple negative | 1 | normal 1 | tumor 1 | Neg. | Neg. | Neg. | 68 | fresh-frozen |
|  |  | 2 | normal 2 | tumor 2 | Neg. | Neg. | Neg. | 85 | fresh-frozen |
|  |  | 3 | normal 3 | tumor 3 | Neg. | Neg. | Neg. | 70 | fresh-frozen |
|  |  | 4 | normal 4 | tumor 4 | Neg. | Neg. | Neg. | 40 | OCT-frozen |
|  |  | 5 | normal 5 | tumor 5 | Neg. | Neg. | Neg. | 85 | OCT-frozen |
|  |  | 6 | normal 6 | tumor 6 |  |  |  |  | OCT-frozen |
|  |  | 7 | normal 7 |  | Neg. | Neg. | Neg. | 80 | OCT-frozen |
|  |  | 8 |  | tumor 8 | Neg. | Neg. | Neg. | 85 | OCT-frozen |
|  | Luminal B-like | 1 | normal 1 | tumor 1 | 70 | 80 | Neg. | 75 | OCT-frozen |
|  |  | 2 | normal 2 | tumor 2 | 60 | 30 | Neg. | 75 | OCT-frozen |
|  |  | 3 | normal 3 |  |  |  |  |  | OCT-frozen |
|  |  | 4 | normal 4 |  |  |  |  |  | OCT-frozen |
|  |  | 5 | normal 5 |  |  |  |  |  | OCT-frozen |
|  |  | 6 |  | tumor 6 | 80 | 60 | Neg. | 60 | OCT-frozen |
|  |  | 7 |  | tumor 7 | 90 | 50 | Neg. | 57 | OCT-frozen |
|  |  | 8 |  | tumor 8 | 95 | 95 | Neg. | 55 | OCT-frozen |

^1^ER: estrogen receptor, PR: progesteron receptor, HER2: human epidermal growth factor receptor 2.

**Table S2.** Summary of ovarian cancer samples.

| **Cancer Type** | **Patient Number** | **Sample Type** | | **Diagnosis** | **Grade** | **Storage** |
| --- | --- | --- | --- | --- | --- | --- |
|  |  | **Normal** | **Tumor** |  |  |  |
| Ovarian cancer | 1 | normal 1 |  | Leiomyoma, NOS |  | FFPE |
|  | 2 | normal 2 |  | Fibroma, NOS |  | FFPE |
|  | 3 | normal 3 |  | Endometrioid carcinoma |  | FFPE |
|  | 4 | normal 4 |  | Endometrioid carcinoma |  | FFPE |
|  | 5 | normal 5 |  | Endometrioid carcinoma |  | FFPE |
|  | 6 |  | tumor 6 | Serous surface cystadenoma, NOS | 3 | FFPE |
|  | 7 |  | tumor 7 | Serous surface papillary carcinoma | 3 | FFPE |
|  | 8 |  | tumor 8 | Serous surface cystadenoma, NOS | 3 | FFPE |
|  | 9 |  | tumor 9 | Serous surface papillary carcinoma | 3 | FFPE |
|  | 10 |  | tumor 10 | Serous surface cystadenoma, NOS | 3 | FFPE |
|  | 11 |  | tumor 11 | Serous surface papillary carcinoma | 3 | FFPE |
|  | 12 |  | tumor 12 | Serous surface papillary carcinoma | 3 | FFPE |
|  | 13 | normal 13 |  | Leiomyoma, NOS |  | FFPE |
|  | 14 | normal 14 |  | Fibroma, NOS |  | FFPE |
|  | 15 | normal 15 |  | Endometrioid carcinoma |  | FFPE |
|  | 16 | normal 16 |  | Endometrioid carcinoma |  | FFPE |
|  | 17 | normal 17 |  | Endometrioid carcinoma |  | FFPE |
|  | 18 |  | tumor 18 | Serous surface papillary carcinoma | 3 | FFPE |
|  | 19 |  | tumor 19 | Serous surface papillary carcinoma | 3 | FFPE |
|  | 20 |  | tumor 20 | Serous surface papillary carcinoma | 3 | FFPE |
|  | 21 |  | tumor 21 | Serous surface papillary carcinoma | 3 | FFPE |
|  | 22 |  | tumor 22 | Serous surface papillary carcinoma | 3 | FFPE |

FFPE: formalin-fixed paraffin-embedded.

**Table S3.** Summary of head and neck samples.

| **Cancer Type** | **Patient Number** | **Sample Type** | | **Diagnosis** | **Grade** | **Storage** |
| --- | --- | --- | --- | --- | --- | --- |
|  |  | **Normal** | **Tumor** |  |  |  |
| Head and neck cancer | 1 | normal 1 | tumor 1 | head and neck squamous cell carcinoma | 3 | FFPE |
|  | 2 | normal 2 |  |  |  | FFPE |
|  | 3 | normal 3 |  |  |  | FFPE |
|  | 4 |  | tumor 4 | head and neck squamous cell carcinoma | 3 | FFPE |
|  | 5 |  | tumor 5 | head and neck squamous cell carcinoma | 1 | FFPE |

FFPE: formalin-fixed paraffin-embedded.

**Table S4.** Summary of prostate cancer samples.

| **Cancer Type** | **Patient Number** | **Sample Type** | | **Diagnosis** | **Storage** |
| --- | --- | --- | --- | --- | --- |
|  |  | **Normal** | **Tumor** |  |  |
| Prostate cancer | 1 | normal 1 | tumor 1 | Adenocarcinoma, NOS | OCT-frozen |
|  | 2 | normal 2 | tumor 2 | Adenocarcinoma, NOS | OCT-frozen |
|  | 3 | normal 3 |  | Adenocarcinoma, NOS | OCT-frozen |
|  | 4 |  | tumor 4 | Adenocarcinoma, NOS | OCT-frozen |

**Table S5.** Summary of cell lines used.

| **Model** | **Cell Line** | **Growth Medium** |
| --- | --- | --- |
| Breast cancer (Luminal-A) | MDA-MB-415 | DMEM+10% NA FBS |
|  | T47D | DMEM+10% NA FBS |
|  | MCF7 | DMEM+10% NA FBS |
| Breast cancer (Triple Negative) | MDA-MB-468 | DMEM/HAM's F12 (1:1) + 10% SA^2^ FBS |
|  | MDA-MB-231 | DMEM+10% NA FBS |
|  | MDA-MB-436 | RPMI1640/HAM's F12 (1:1)+10% SA FBS |
|  | SUM149 | RPMI1640/HAM's F12 (1:1)+10% SA FBS |
| Breast | MCF10A | MEBM+bovine pituitary extract 3 mg/ml+hydrocortisone 0.5 mg/ml+hEGF 10 ug/ml+insulin 5 mg/ml+100 ng/ml cholera toxin |
|  | MCF12 | DMEM/HAM's F12 (1:1) + hEGF 20 ng/ml, 100 ng/ml cholera toxin, 0.01 mg/ml insulin+hydrocortisone 0.5 mg/ml+5% horse serum |
|  | HBL-100 | McCoy'S 5A+ 10% NA FBS |
| Prostate (HPV infected) | PZ-HPV-7 | Keratinocyte Serum Free Medium (K-SFM) +0.05 mg/ml BPE +5 ng/ml EGF |
|  | RWPE-1 | Keratinocyte Serum Free Medium (K-SFM) +0.05 mg/ml BPE +5 ng/ml EGF |
|  | WPE1-NA22 | Keratinocyte Serum Free Medium (K-SFM) +0.05 mg/ml BPE +5 ng/ml EGF |
|  | CA-HPV10 | Keratinocyte Serum Free Medium (K-SFM) +0.05 mg/ml BPE +5 ng/ml EGF |
| Prostate cancer | DU-145 | DMEM+10% SA FBS |
|  | LNCaP | RPMI+10% NA FBS |
|  | PC3 | Ham's F12+10% NA-FBS |

^1^NA: North American, ^2^SA: South American.


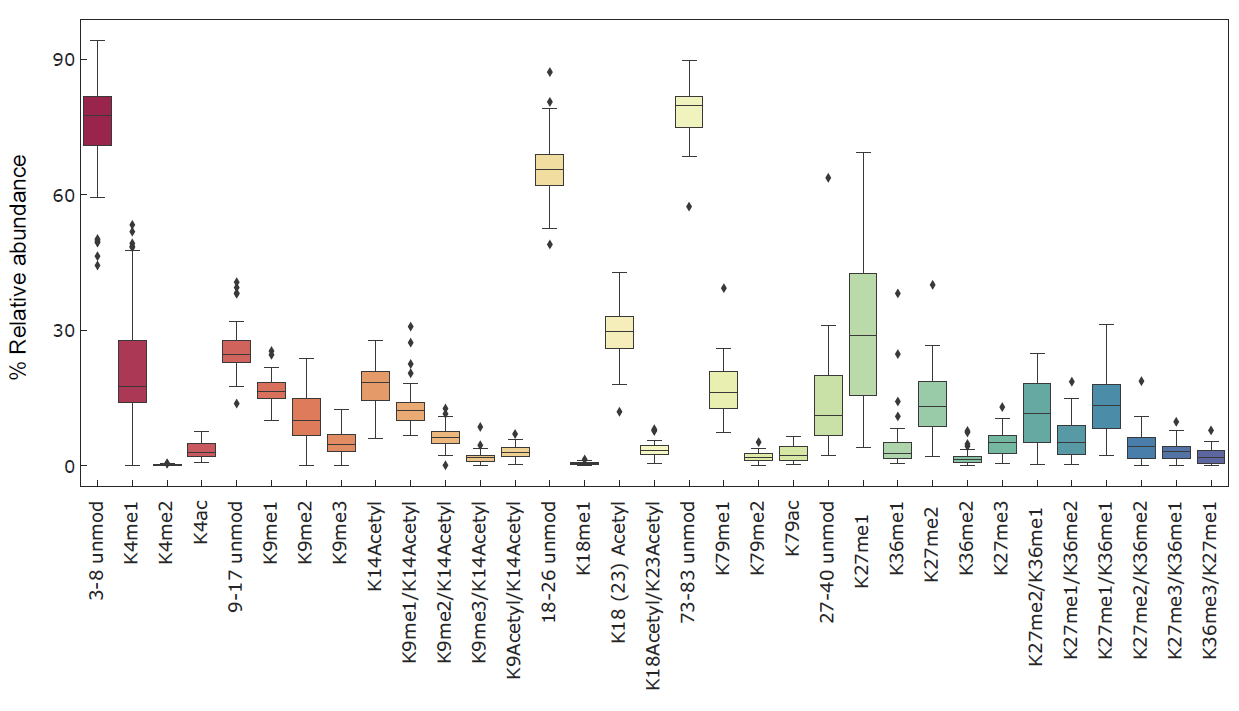


**Figure S1.** Histogram representation of the % relative abundance values shown in Figure 1.

**
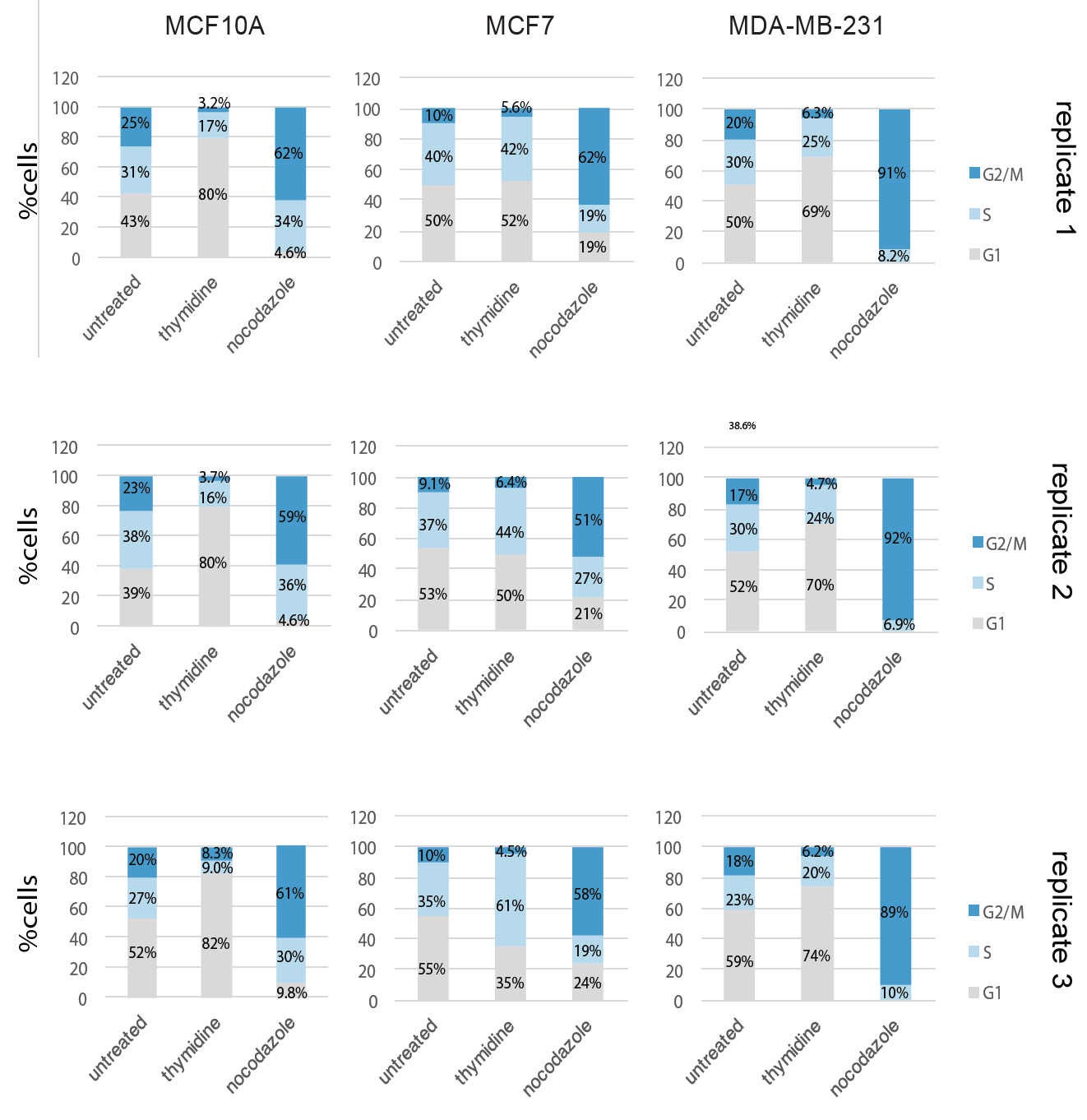
Figure S2.** Breast cancer cell line synchronization (1). Histograms showing the percentages of cells in G1, S and G2/M phases for MCF10A, MCF7 and MDA-MB-231 breast cancer cells untreated, treated with thymidine or nocodazole. Three biological replicates are shown.


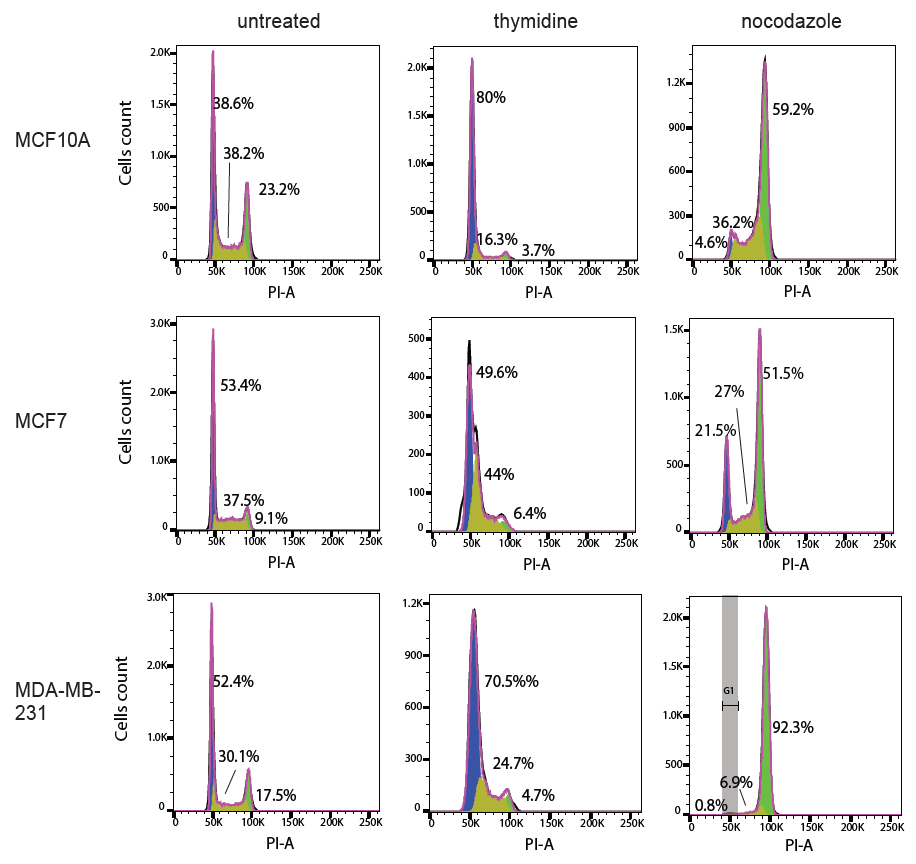


**Figure S3.** Breast cancer cell line synchronization (2). Cell cycle distributions of MCF10A, MCF7 and MDA-MB-231 cells untreated, treated with thymidine or nocodazole for one biological replicate. Cell cycle distributions were obtained following FACS analysis of DNA area of propidium iodide-stained cells.


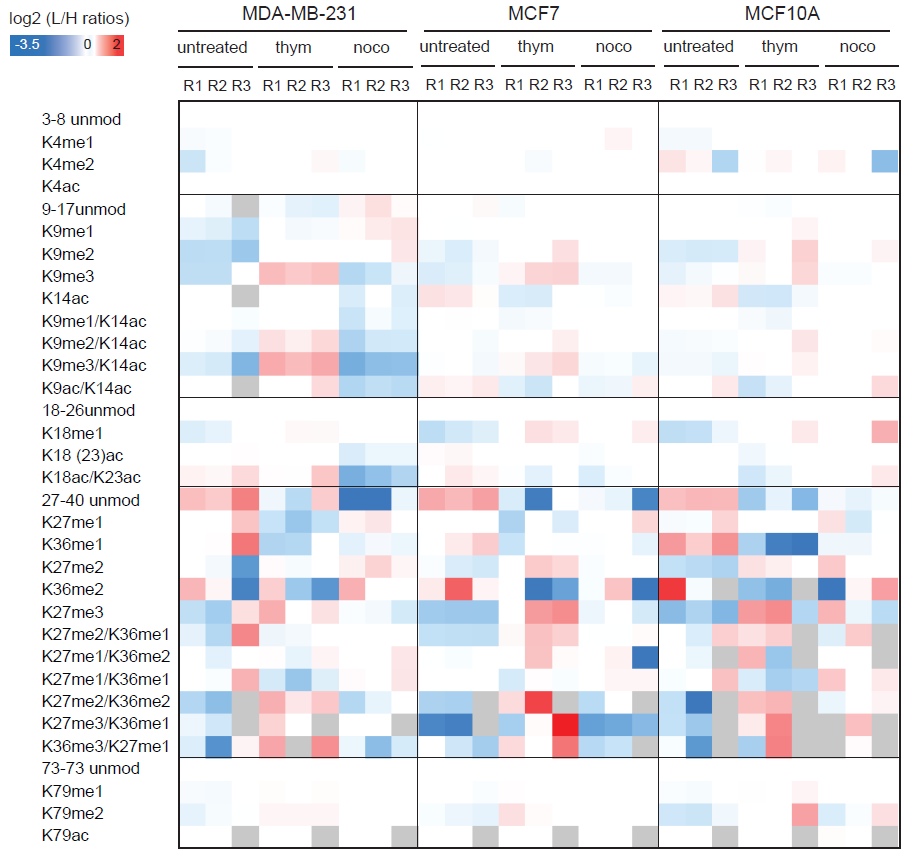


**Figure S4.** Histone PTM profiling of cells synchronized in different phases of the cell cycle. Heatmap display of log2 transformed ratios obtained for the indicated histone H3 PTMs for MCF10A, MCF7 and MDA-MB-231 cells untreated, treated with thymidine or treated with nocodazole (three biological replicates). L/H relative abundances ratios obtained with the super-SILAC strategy (light channel: breast cell line, heavy channel: spike-in super-SILAC standard), normalized over the average value for each cell line are shown. Ratios between nocodazole- and thymidine-treated cells are shown in Figure 2B.


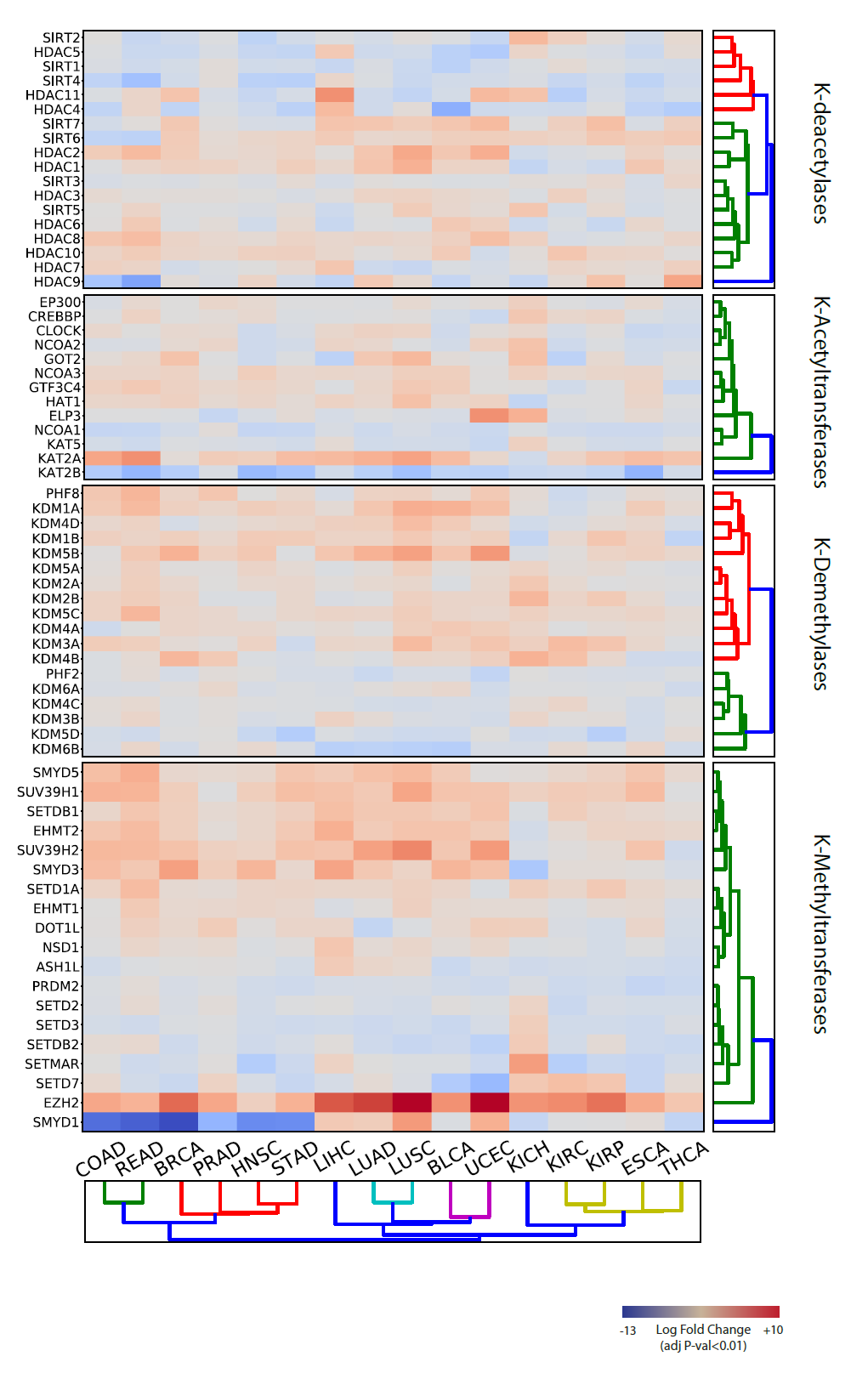


**Figure S5.** Heatmap representation of the data shown in Figure 3.


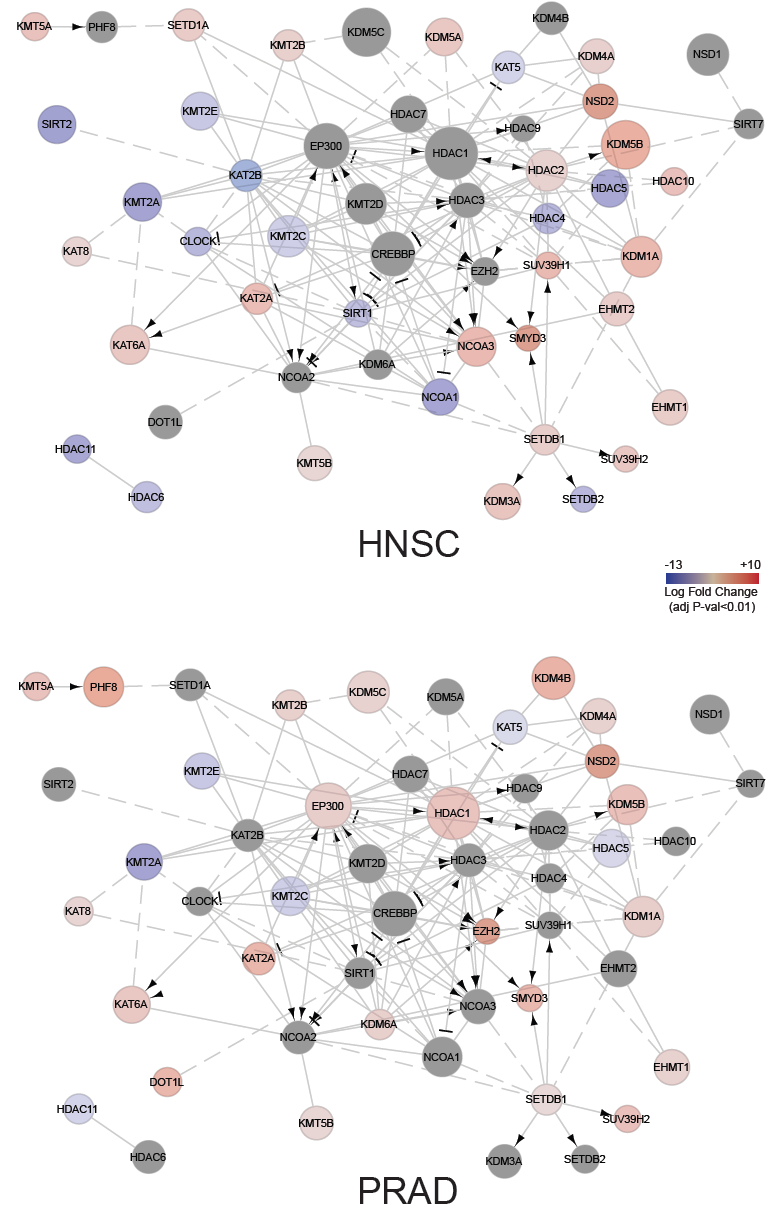


**Figure S6.** HME interaction networks for HNSC (Head and Neck squamous cell carcinoma) and PRAD (Prostate adenocarcinoma), where the red and blue colors indicate up- or down-regulation in tumors compared with normal tissues, and node diameters are proportional to RPKM base mean from DESEQ2 analysis.

**Figure S7.** (separate PDF file). Frequency and type of mutations of individual HMEs in the TCGA PanCan 2018 cohort. Stacked bar plot summarizing the frequency and type of mutations of individual HMEs in the TCGA PanCan 2018 cohort (minimum % of altered cases=1). Whether the genes have been reported as oncogenes and/or tumor suppressor genes (TSG) in at least one tumor type in the Cancer Census Genes is indicated. Simple mutations are colored in green, fusions in purple, amplifications in red, deep deletions in blue, and multiple alterations in grey. Amplifications in one/more tumor types are suggestive of a potential oncogenic role, while deletions are suggestive of a potential tumor suppressor role.
