# Supplementary material for: Profiling of Epigenetic Features in Clinical Samples Reveals Novel Widespread Changes in Cancer
Source: Cancers (Basel). 2019 May 24;11(5):723. doi: 10.3390/cancers11050723 (PMC6562406; doi:10.3390/cancers11050723)

Acetyltransferases

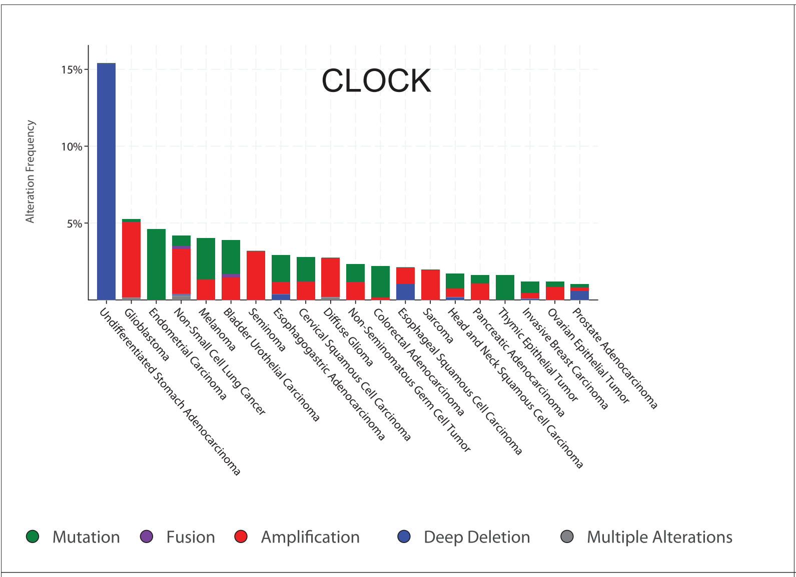

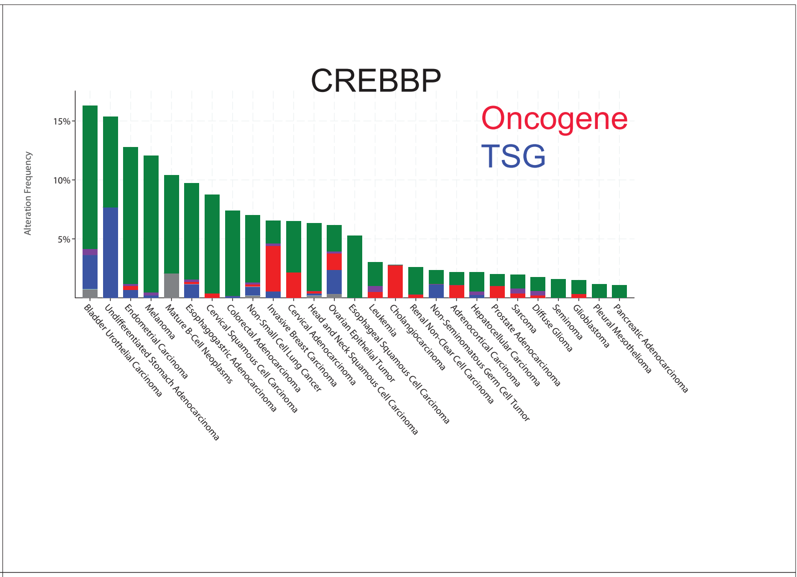

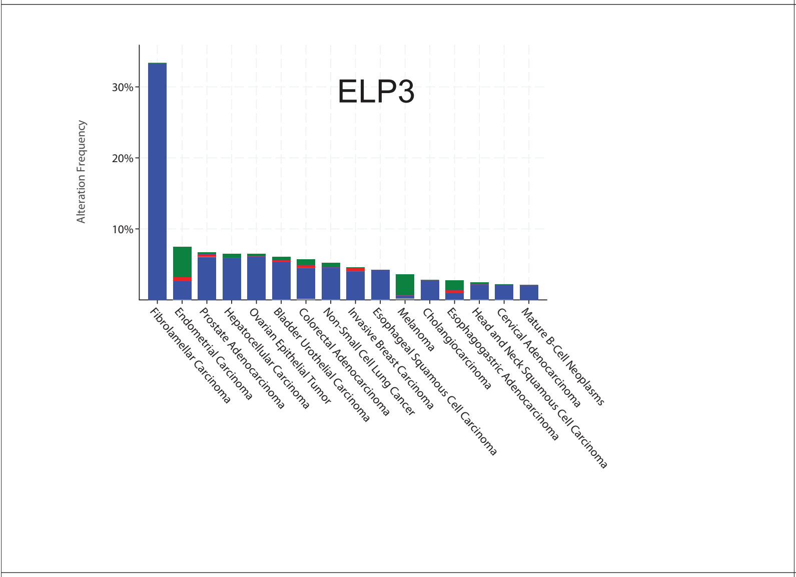

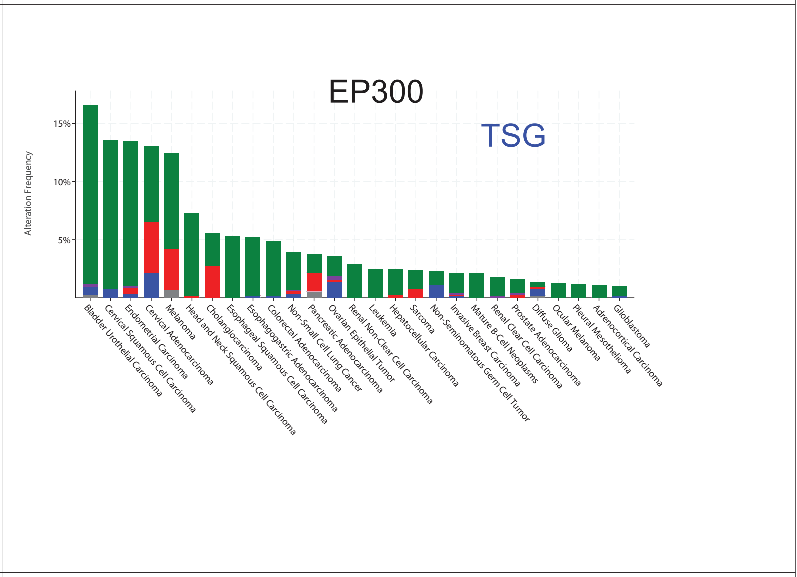

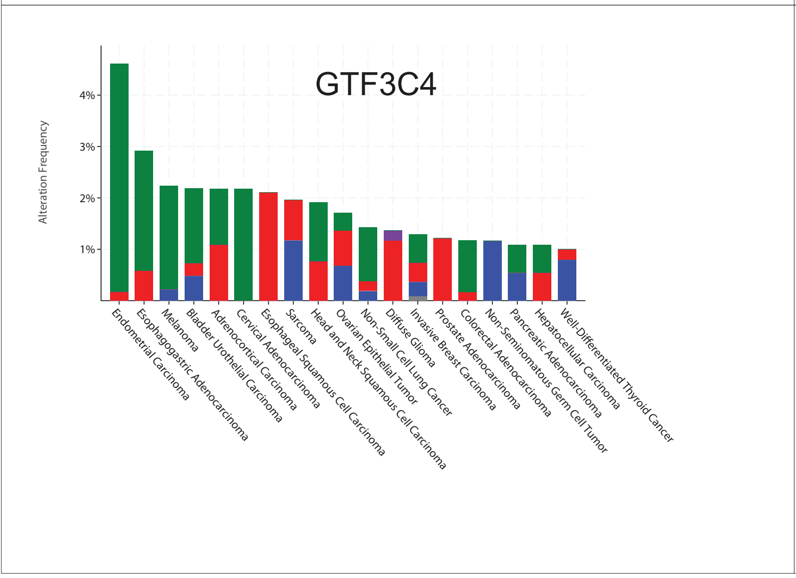

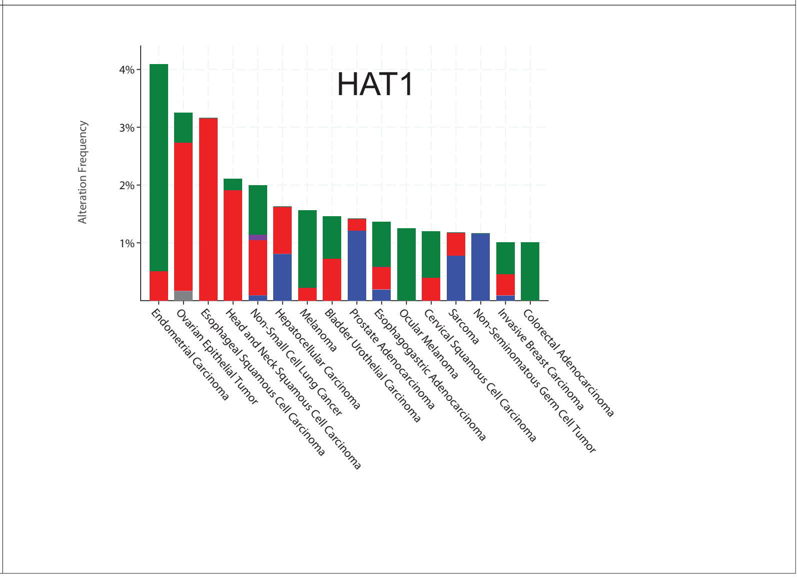

Acetyltransferases

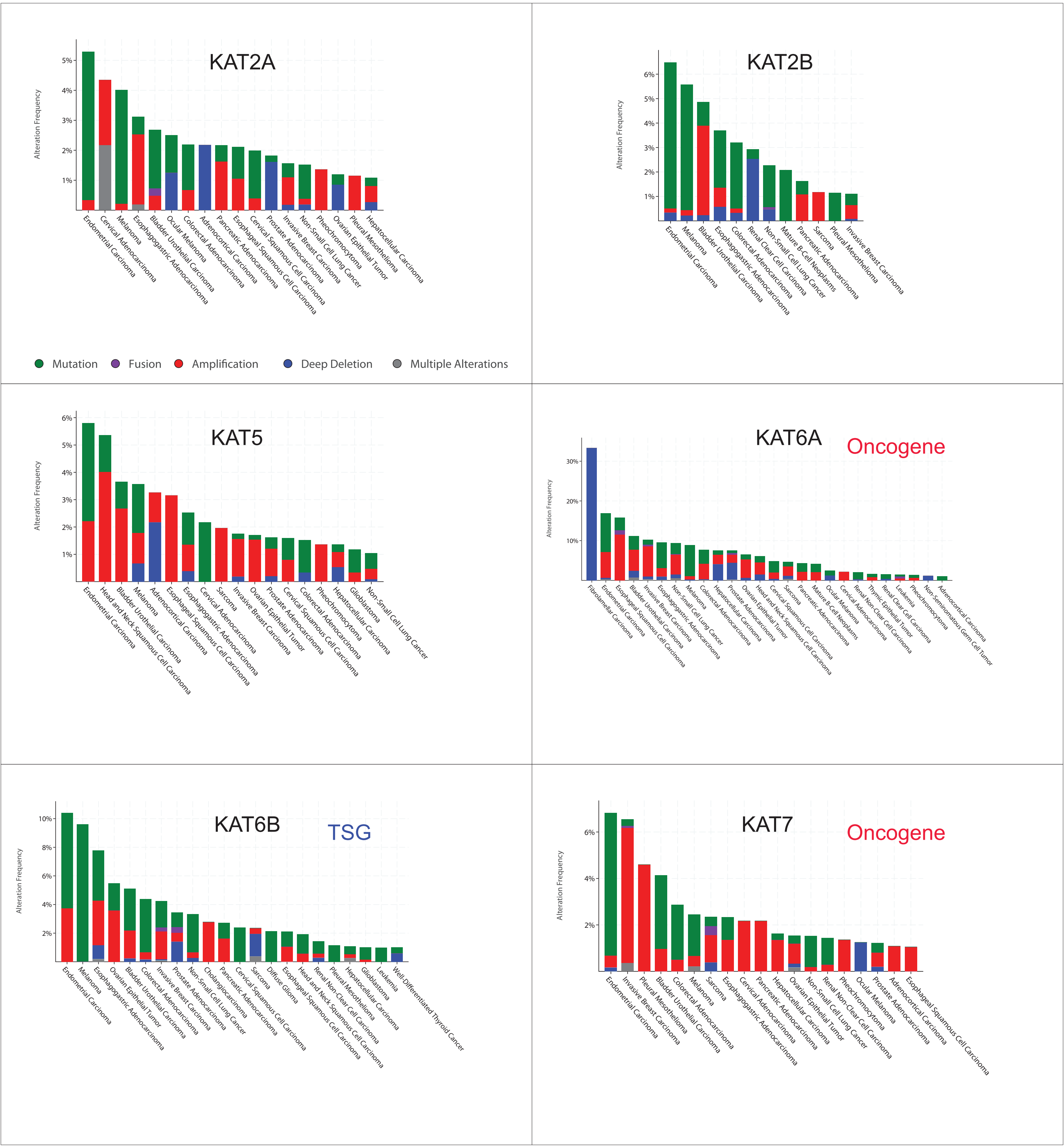

Acetyltransferases

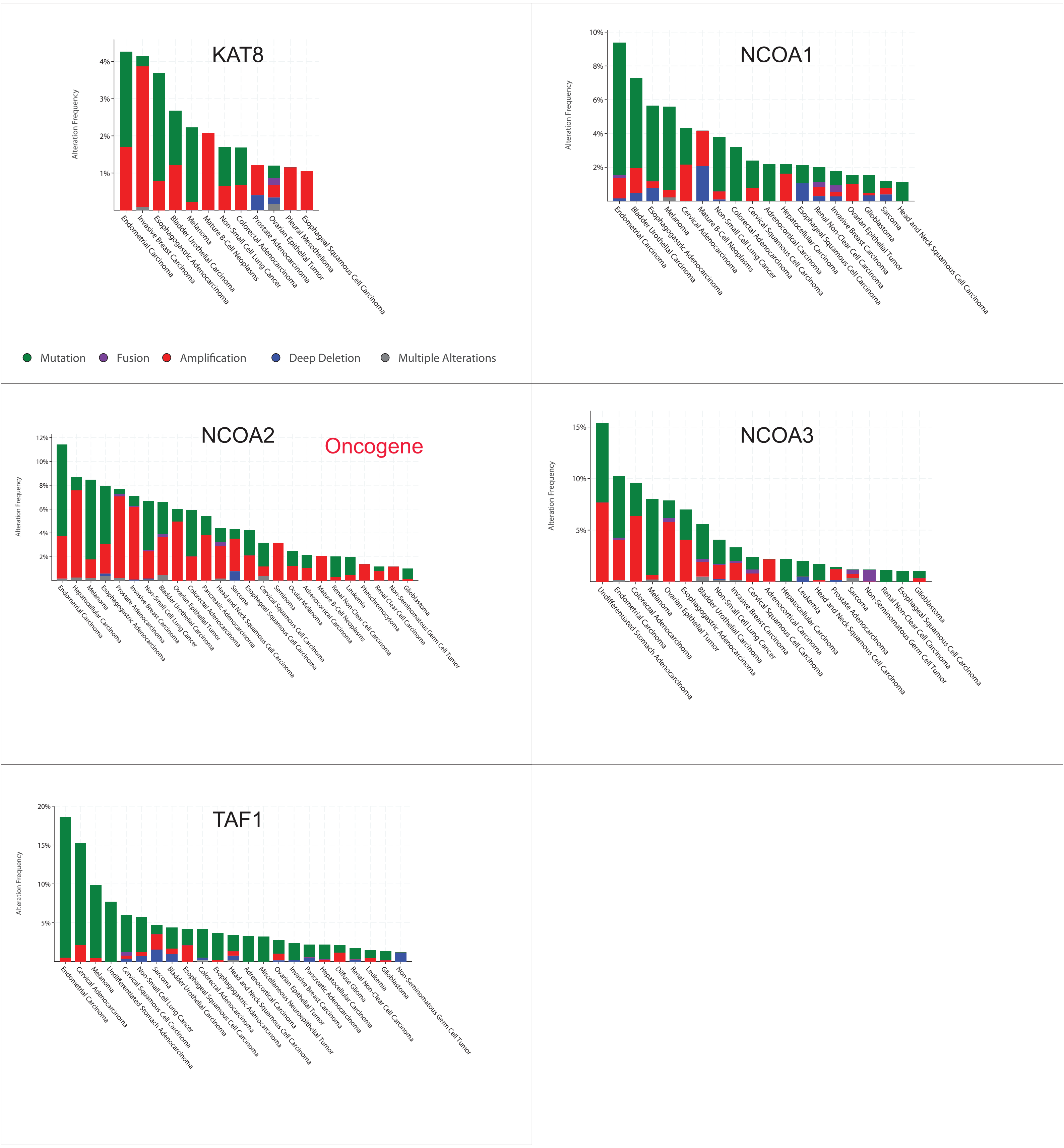

Deacetylases

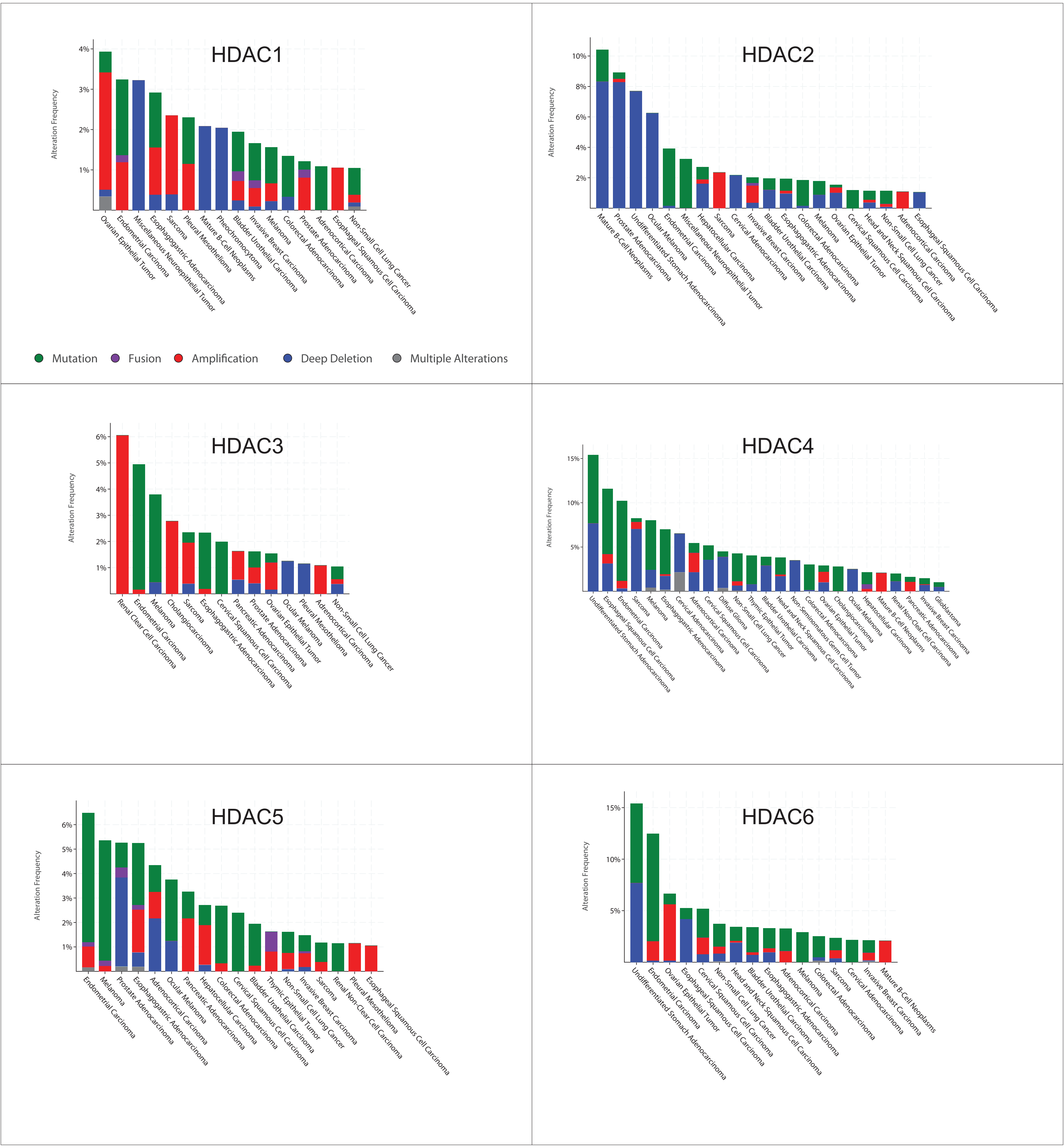

Deacetylases

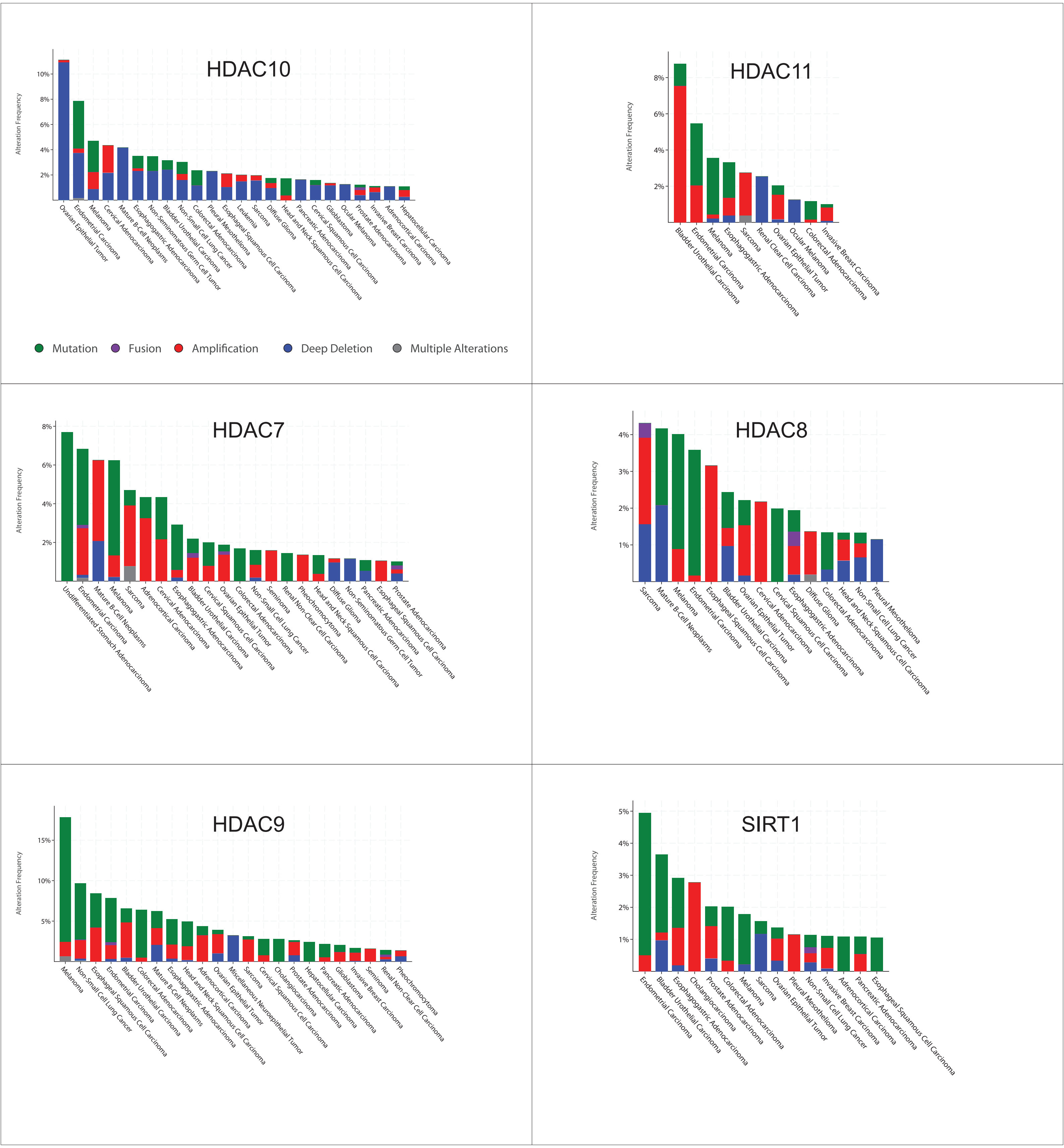

Deacetylases

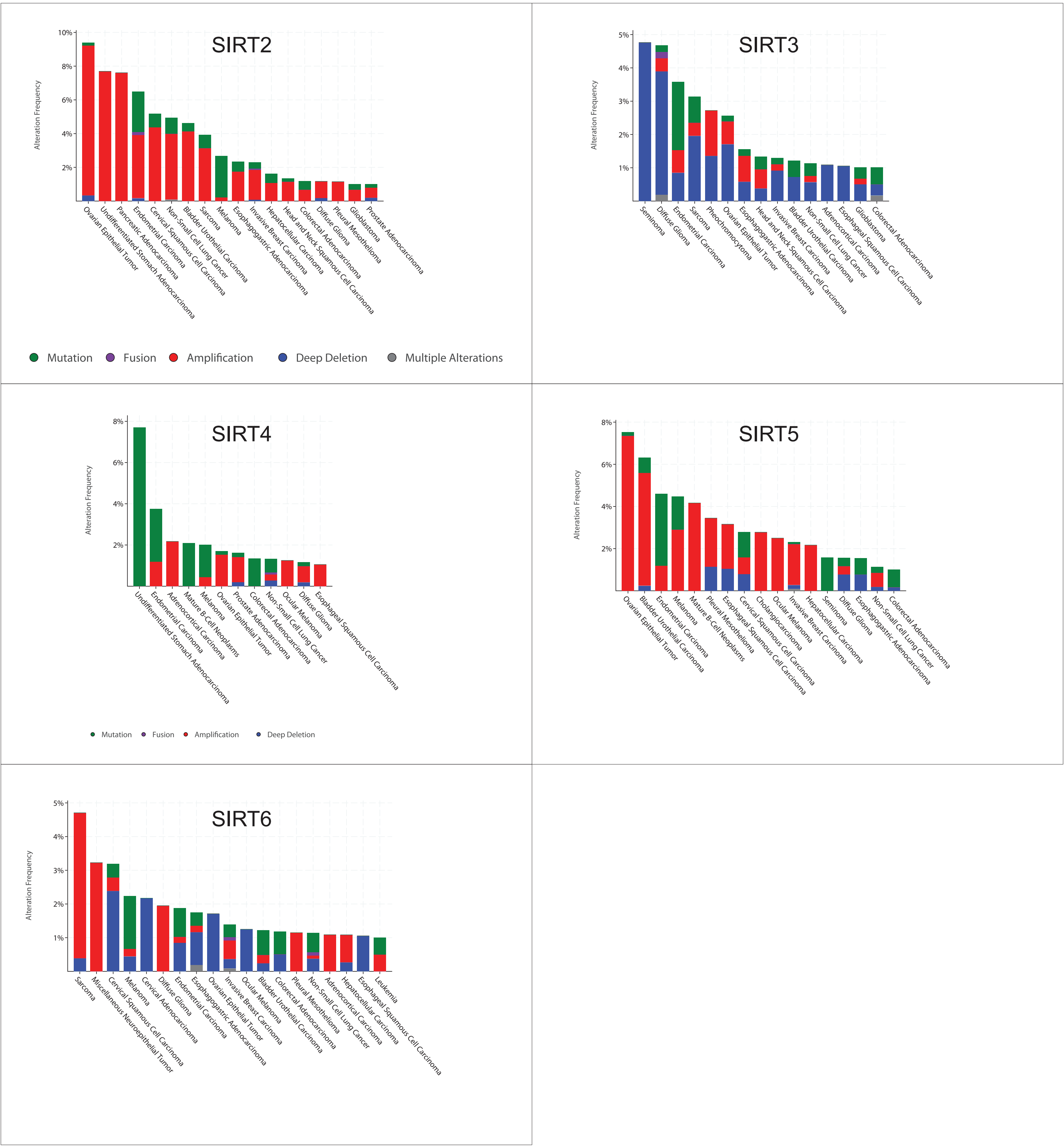

Methyltransferases

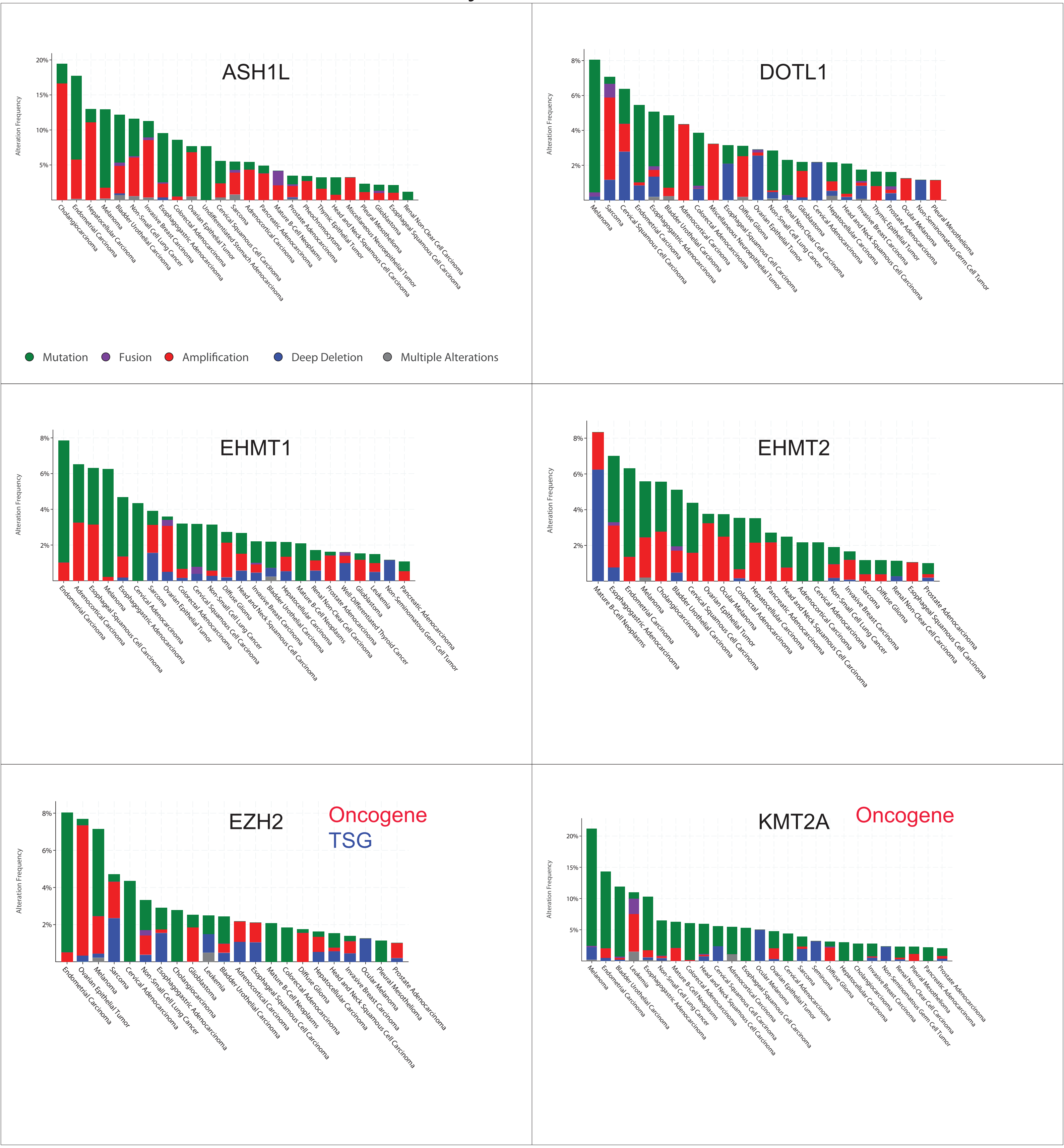

Methyltransferases

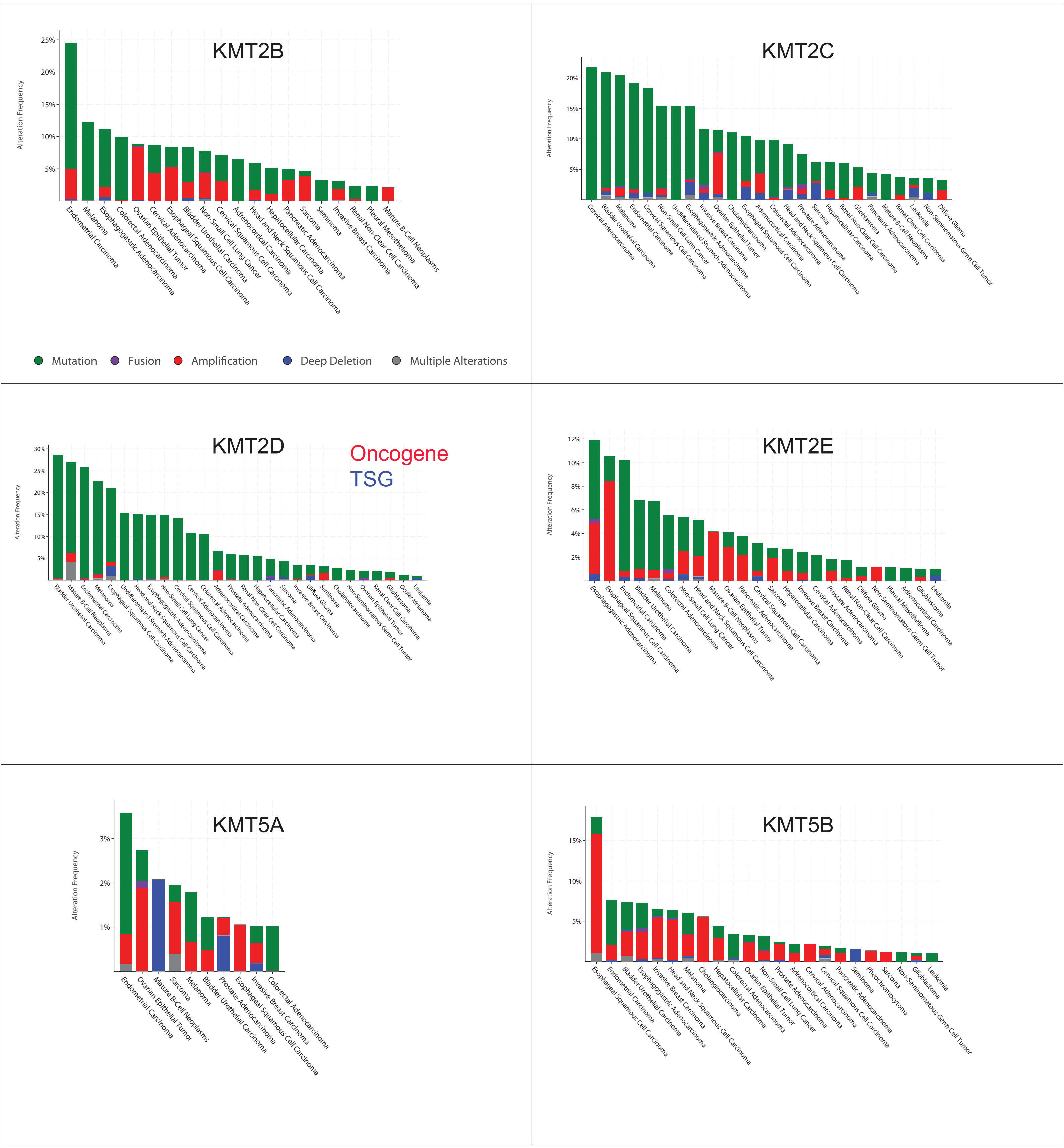

Methyltransferases

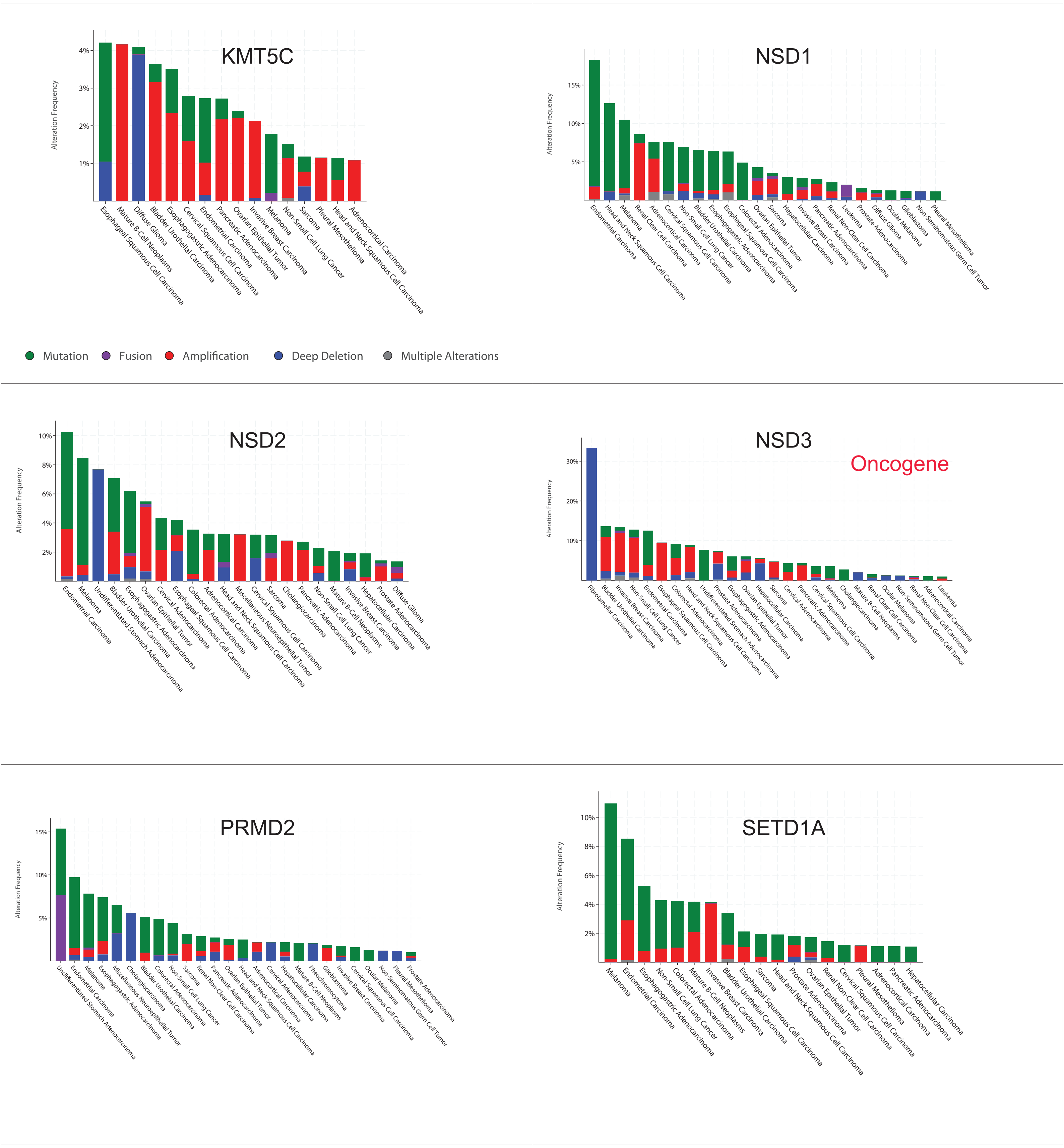

Methyltransferases

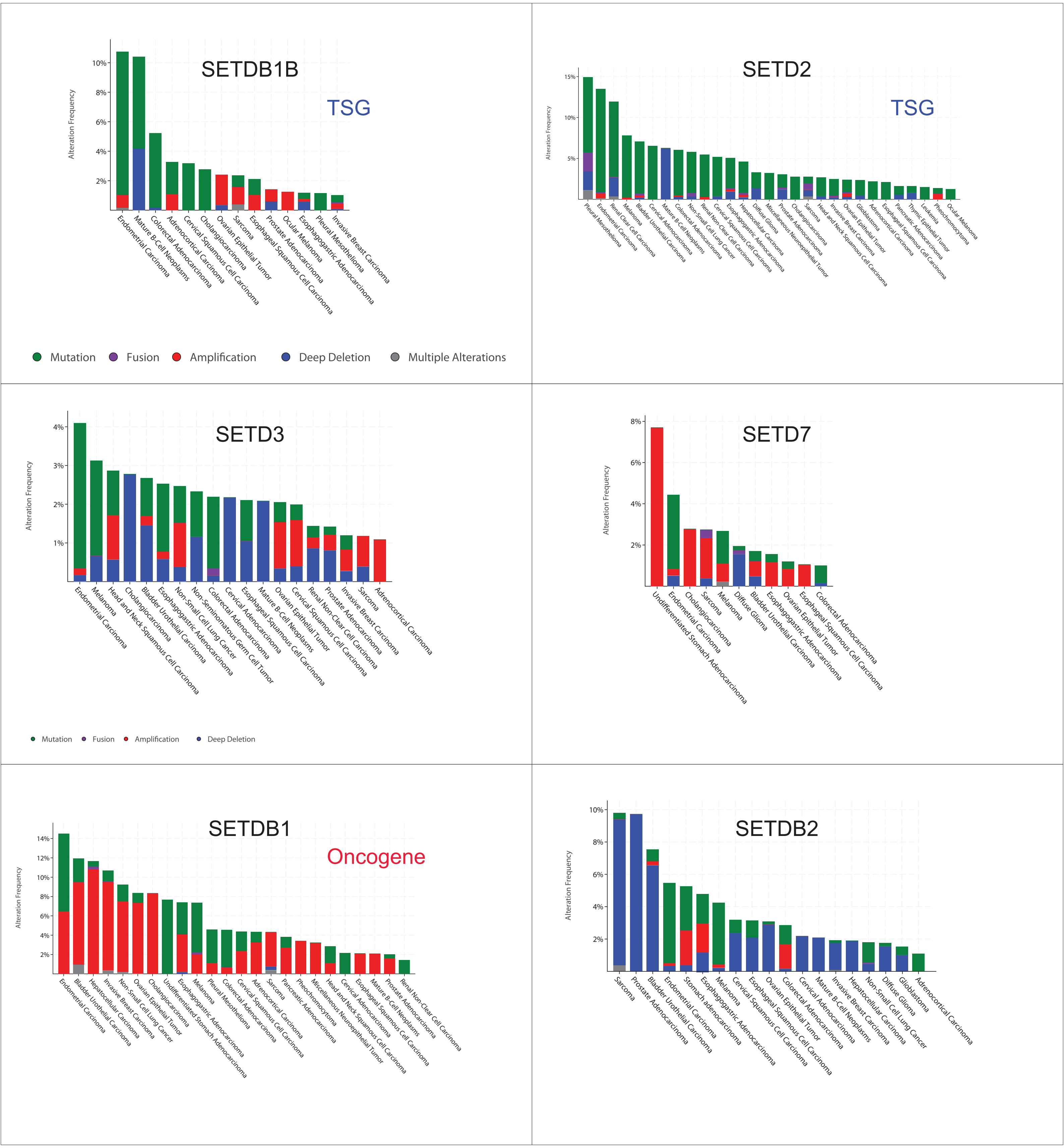

Methyltransferases

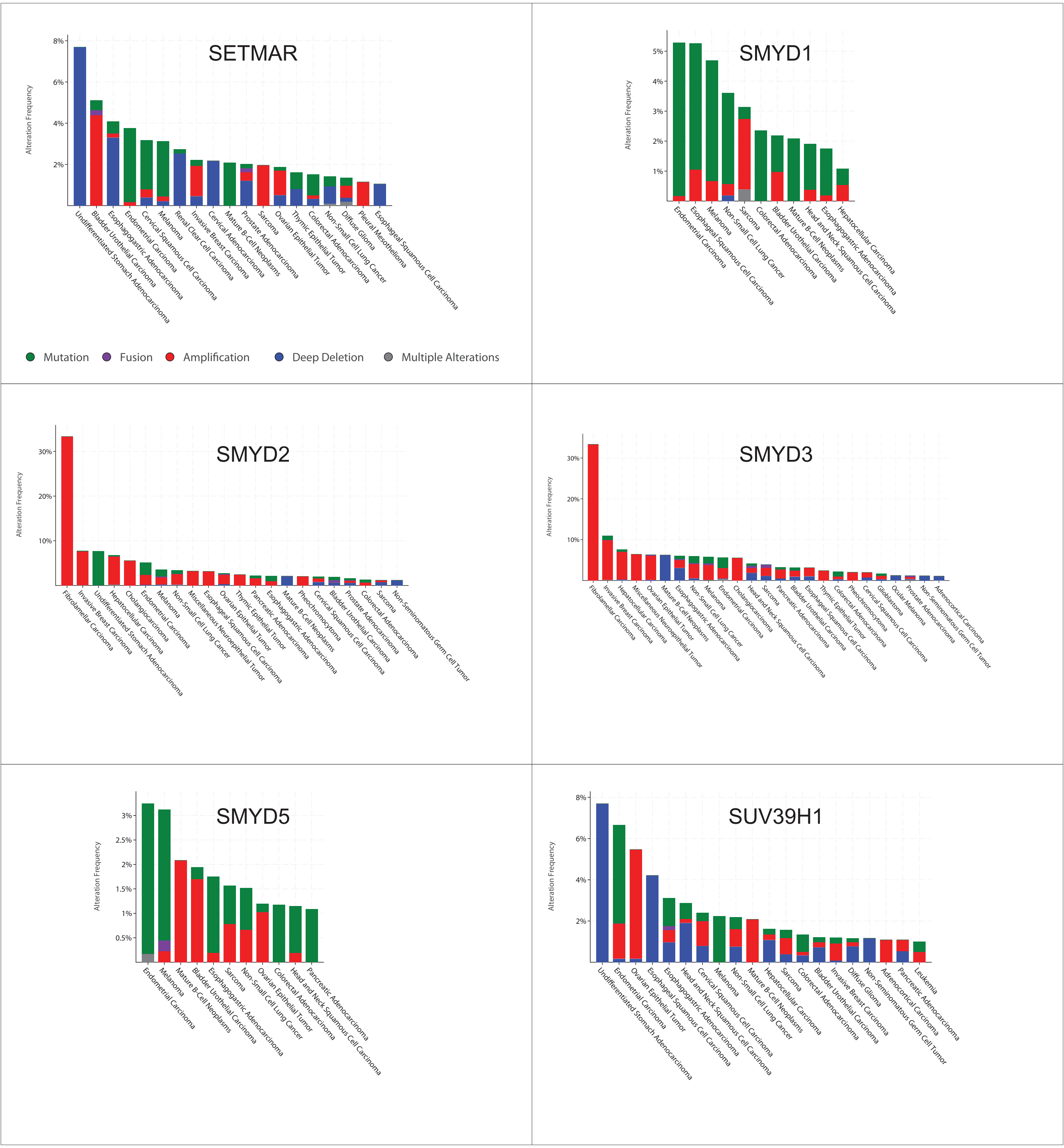

Methyltransferases

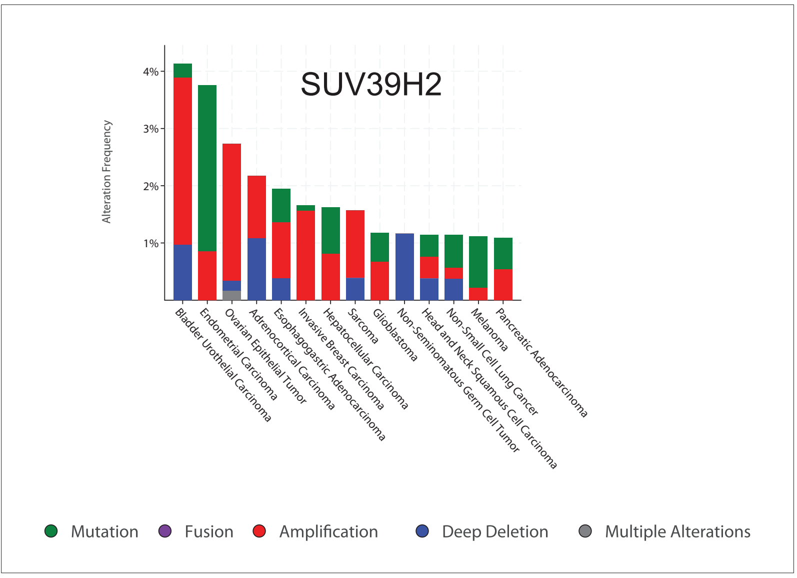

Demethylases

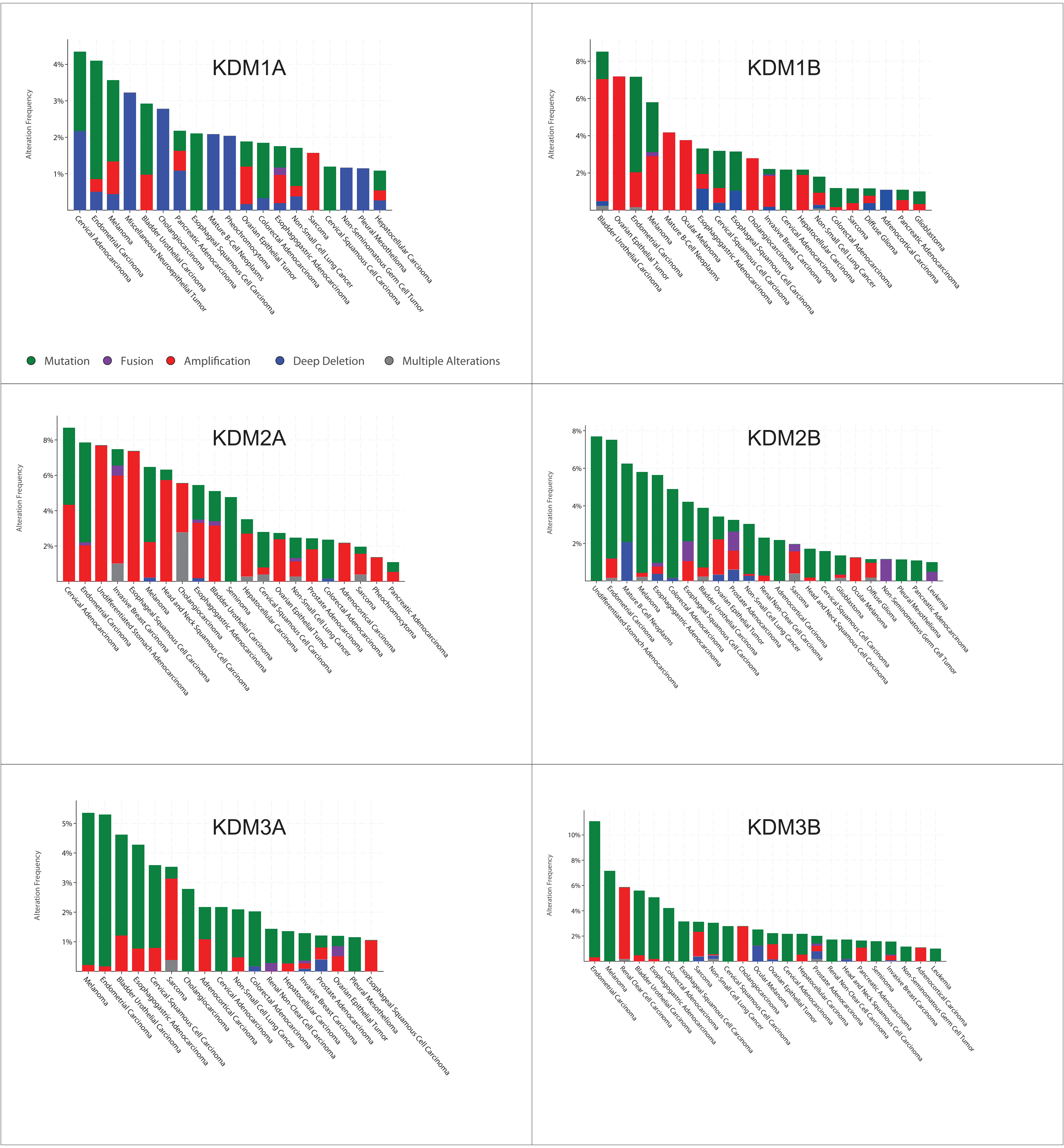

Demethylases

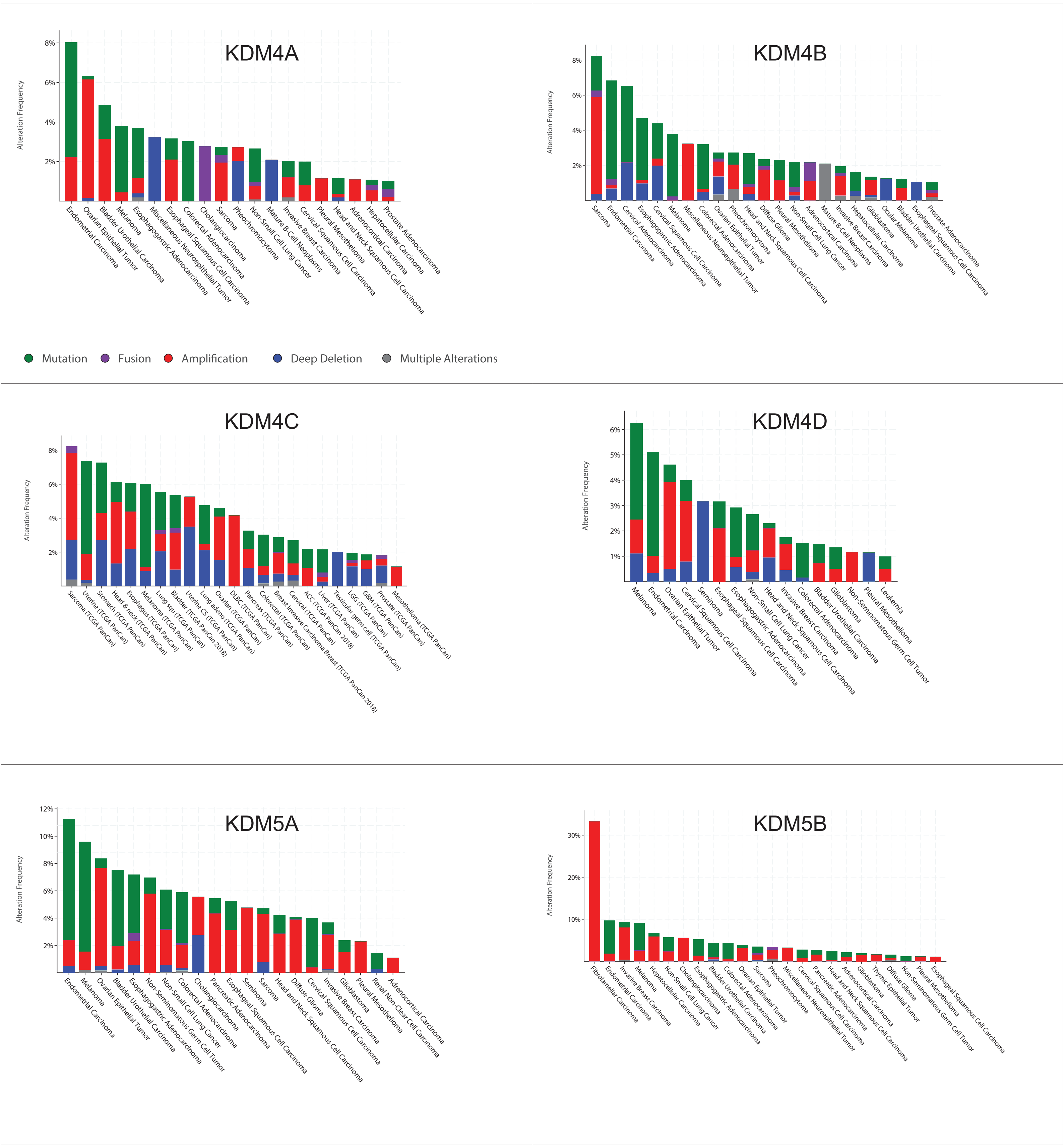

Demethylases

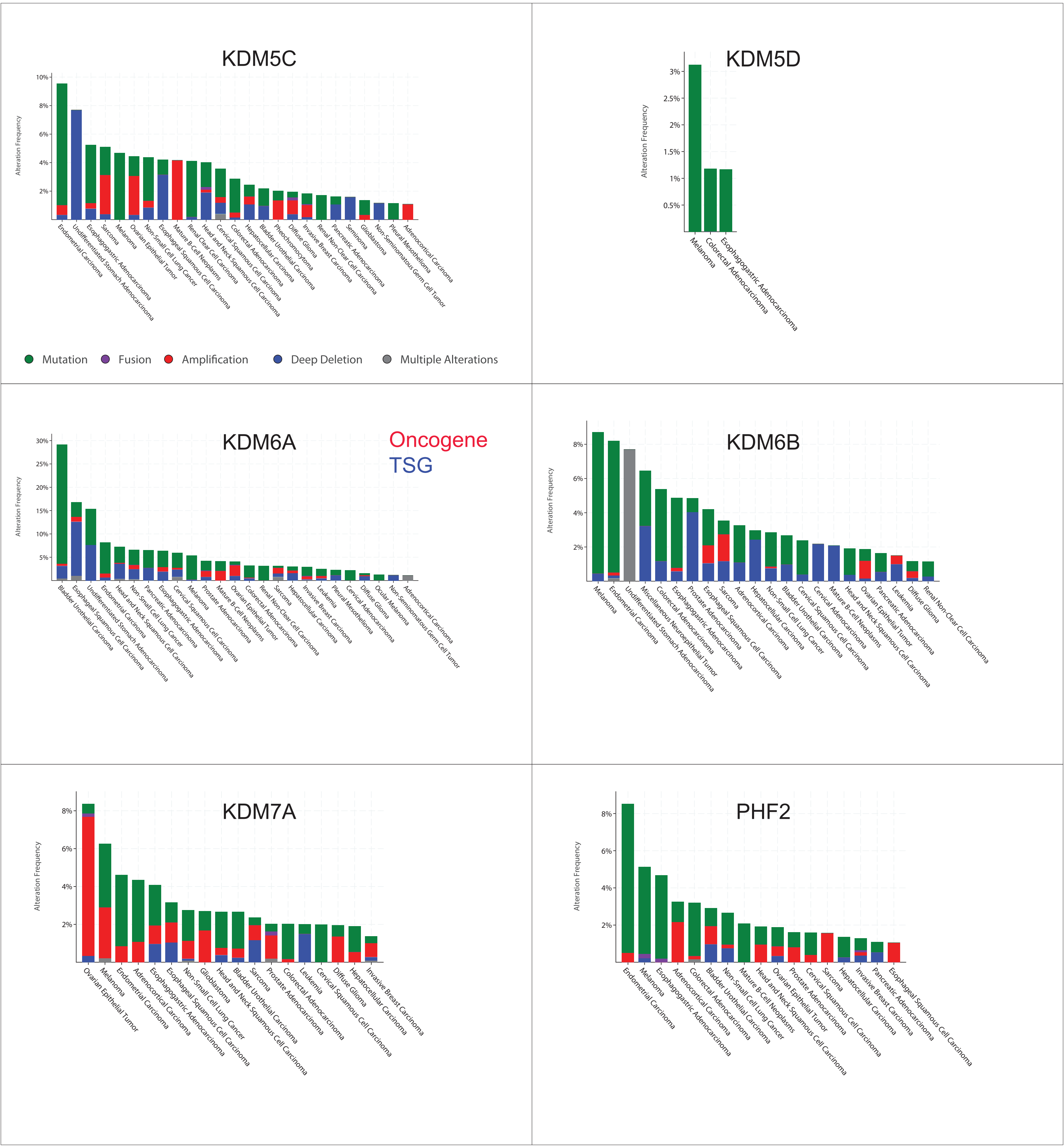

Demethylases

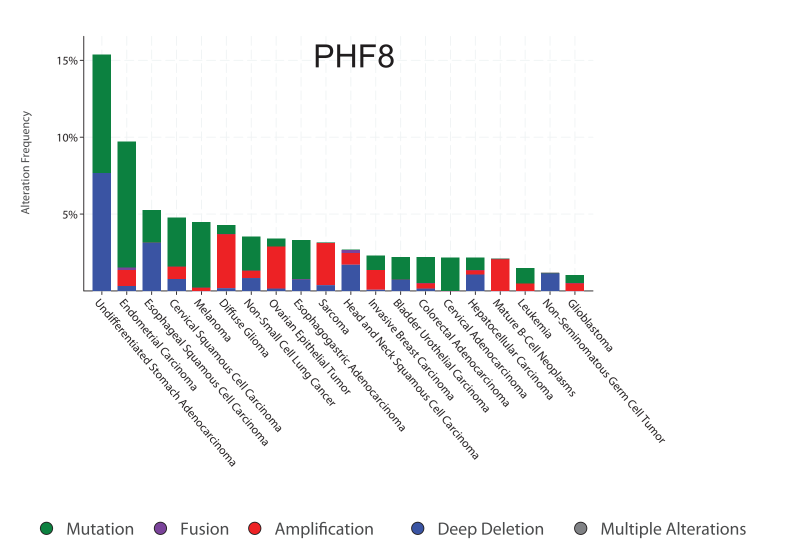

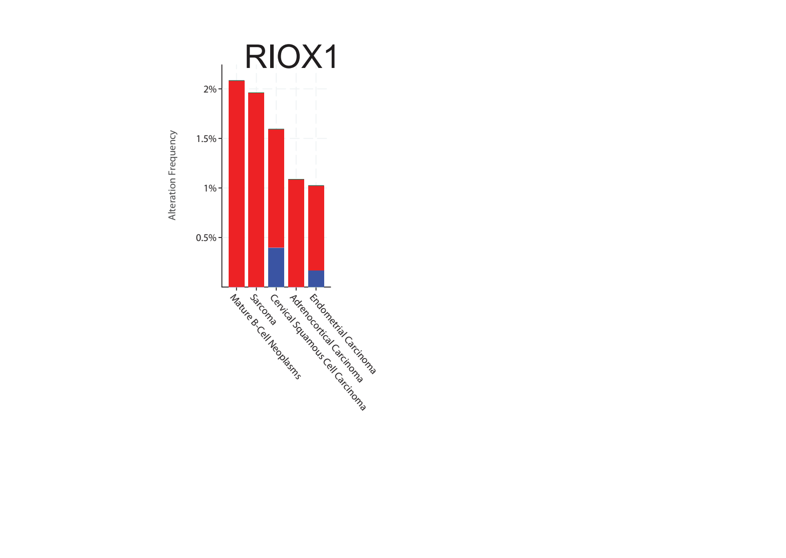

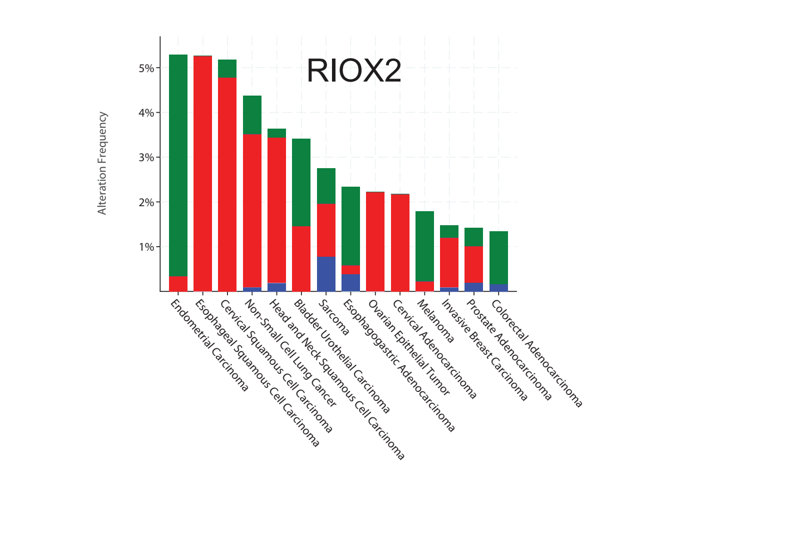

Supplement: Supplementary file 1 [file cancers-11-00723-s001.zip › cancers-484721-supplementary/Figure S7.pdf]
